# Disentangling the multiple links between renal dysfunction and cerebrovascular disease

**DOI:** 10.1136/jnnp-2019-320526

**Published:** 2019-09-11

**Authors:** Dearbhla Kelly, Peter Malcolm Rothwell

**Affiliations:** Centre for the Prevention of Stroke and Dementia, Nuffield Department of Clinical Neurosciences, University of Oxford, Oxford, UK

**Keywords:** chronic kidney disease, stroke, hypertension, dialysis, dementia

## Abstract

Chronic kidney disease (CKD) has a rapidly rising global prevalence, affecting as many as one-third of the population over the age of 75 years. CKD is a well-known risk factor for cardiovascular disease and, in particular, there is a strong association with stroke. Cohort studies and trials indicate that reduced glomerular filtration rate increases the risk of stroke by about 40% and that proteinuria increases the risk by about 70%. In addition, CKD is also strongly associated with subclinical cerebrovascular abnormalities, vascular cognitive impairment and dementia. The mechanisms responsible for these associations are currently unclear. CKD is associated with traditional risk factors such as hypertension, diabetes mellitus and atrial fibrillation, but non-traditional risk factors such as uraemia, oxidative stress, mineral and bone abnormalities, and dialysis-related factors, such as changes in cerebral blood flow or cardiac structure, are also postulated to play a role. Kidney disease can also impact and complicate the treatments used in acute stroke and in secondary prevention. In this review, we will outline our current understanding of the epidemiology and pathophysiology of cerebrovascular disease in CKD.

## Introduction

Chronic Kidney Disease (CKD) affects as many as 8-16% of the population worldwide.^1^ It is an increasingly important global health burden, mainly because it is an established risk factor for cardiovascular disease. Compared with the general population, cardiovascular diseases such as stroke are more frequent and severe, and are often undertreated in people with CKD. Even in less advanced stages of CKD, cardiovascular mortality is still much higher than the incidence of kidney failure.^2^ Despite improvements in cardiovascular disease survival in the general population, the rate of progress in patients with CKD, especially in those who are dialysis dependent, has lagged behind.^3^ In particular, CKD contributes to the risk and severity of stroke, subclinical cerebrovascular abnormalities and vascular dementia.^4^

In this review, we will explore the risk, severity and mechanisms for stroke in CKD. Firstly, we will discuss the clinical associations between renal and cerebrovascular diseases, including ischaemic stroke, small-vessel disease (SVD), haemorrhagic stroke, vascular cognitive impairment and dementia. Secondly, we will highlight the mechanisms of susceptibility and injury, including shared pathophysiology and risk factors, secondary consequences of renal dysfunction, and diseases that can cause both CKD and stroke.

## Associations between CKD and cerebrovascular disease

### Stroke risk

There is conflicting evidence about whether CKD, specifically low estimated glomerular filtration rate (eGFR), is a risk factor for stroke independent of traditional cardiovascular risk factors. In a meta-analysis of 22 634 people from four population-based longitudinal studies, individuals with an eGFR of <60 mL/min/1.73 m^2^ had an incidence rate of 10.3 stroke events per 1000 person-years compared with 3.4 events per 1000 person-years in those without; however, this apparent excess risk of stroke was no longer statistically significant after adjusting for conventional vascular risk factors (HR 1.17, 95% CI 0.95–1.44; p=0.13).^5^

However, this attenuation of risk association may simply have been the result of inadequate statistical power, as a larger meta-analysis of 33 prospective studies with 284 672 participants experiencing 7863 stroke events reported that patients with an eGFR of <60 mL/min/1.73 m^2^ had a 43% increased risk of stroke in a pooled multivariate-adjusted analysis that adjusted for conventional vascular risk factors,^6^ with an increase in both ischaemic and haemorrhagic strokes.

Similarly, in the most recent meta-analysis of 83 studies (over two million participants), there was an inverse linear relationship between eGFR and the risk of stroke, with risk of stroke increasing 7% for every 10 mL/min/1.73 m^2^ decrease in glomerular filtration rate (GFR).^7^ A 25 mg/mmol increase in the albumin to creatinine ratio (ACR) was associated with a 10% increased risk of stroke. As in the previous meta-analysis, the results were consistent across stroke subtypes, by sex or in subgroup strata with varying prevalence of vascular risk factors (hypertension, diabetes and smoking), suggesting an independent relationship between CKD and stroke risk. Stroke risk increased linearly and additively with declining GFR and increasing albuminuria.

There is a particularly strong and independent association between proteinuria and stroke. In a meta-analysis of 10 cohort studies (140 231 participants, 3266 strokes), participants with proteinuria had a 71% greater risk of stroke compared with those without (95% CI 1.39% to 2.10%).^8^ The risk of stroke remained high after adjustment for other cardiovascular risk factors, but there may be residual confounders. There is a dose–response relationship with higher levels of albuminuria conferring greater stroke risk. In another meta-analysis, patients with macroalbuminuria had a higher risk of incident stroke (relative risk (RR) 2.65, 95% CI 2.25 to 3.14) compared to that of those with microalbuminuria (RR 1.58, 95% CI 1.39 to 1.80).^9^

### Stroke outcomes

Patients with CKD tend to suffer more severe strokes with worse functional outcomes, morbidity and mortality. This was demonstrated by the Fukuoka Stroke Registry multicentre study of 3778 patients with first-ever ischaemic stroke.^10^ On admission, the National Institutes of Health Stroke Scale (NIHSS) scores of patients with CKD were significantly higher than those without CKD. After adjustment for possible confounding factors, including age, baseline NIHSS score, cardioembolic aetiology, blood pressure (BP) on admission, history of hypertension or diabetes, thrombolytic therapy and infectious complications, patients with CKD had a 49% (95% CI 17% to 89%) greater risk of neurological deterioration during their hospitalisation (defined as a ≥2-point increase in the NIHSS score), a 138% (95% CI 61% to 257%) greater risk of in-hospital mortality and a 25% (95% CI 5% to 48%) greater risk of a Modified Rankin Scale score of ≥2 at discharge.

Functional outcomes and stroke mortality are worse in advanced CKD. In a cohort study of 232 236 patients, patients with an eGFR of <15 mL/min/1.73 m^2^ (not on dialysis) had a 2.5 times greater risk of in-hospital mortality after acute ischaemic stroke compared with those with normal renal function, even after adjusting for comorbidities and initial NIHSS score.^11^ This subgroup of patients also had much lower rates of functional independence and discharge to home.

### Cerebral SVD and CKD

CKD is also highly prevalent in patients with SVD. In a single-centre study of survivors of acute ischaemic stroke, patients with silent lacunar infarction, white matter lesion (WML) or cerebral microbleed (CMB) had significantly lower eGFR than those without such lesions (60.4±34.8 vs 87.5±28.4 mL/min/1.73 m^2^, 60.5±37.1 vs 73.9±33.3 mL/min/1.73 m^2^ and 57.6±33.3 vs 73.9±32.9 mL/min/1.73 m^2^, respectively).^12^ Even with adjustment for age and vascular risk factors, the OR for the presence of SVD and each subtype increased inversely with eGFR, with the highest prevalence in more advanced CKD. Patients with CKD with each SVD subtype had lower survival than those without such lesions, and in particular, the presence of WML was an independent risk factor for cardiovascular death. Stratified meta-analyses consistently show significant associations with low eGFR across different SVD subtypes, with a nearly threefold increased risk of silent cerebral infarctions and CMB.^13^ From the Cilostazol versus Aspirin for Secondary Ischemic Stroke Prevention (CASISP) study,^14^ decreased baseline eGFR, CKD progression and history of hypertension were independently associated with the presence of deep or infratentorial CMB at follow-up, but not lobar CMB.

There appears to be a particular pattern of white matter disease in CKD. In a Chinese group of 1632 patients, CKD was independently associated with the severity of periventricular hyperintensities (PVHs) but not deep subcortical white matter in patients with acute ischaemic stroke.^15^ The severity of white matter hyperintensities (WMHs) was evaluated using the Fazekas scale. Each 30 mL/min/1.73 m^2^ increase in eGFR was associated with a 75% risk of the presence of degree 3 of PVH compared with degree 0. These findings highlight potentially differential pathological mechanisms underlying white matter changes in CKD.

The Oxford Vascular Study highlighted an age-specific association between SVD and CKD in 1080 patients with transient ischaemic attack (TIA) and ischaemic stroke.^16^ CKD was associated with total SVD score but only in a younger population (<60 years) (OR 3.97, 95% CI 1.69 to 9.32; p=0.002). This association was diminished but remained after adjustment for age, sex, history of hypertension or diabetes, and premorbid average systolic BP (OR 3.11, 95% CI 1.21 to 7.98; p=0.018). The strong association between CKD and SVD at younger ages could indicate a shared genetic vulnerability to premature cardiovascular disease, accentuated by acquired risk factors such as hypertension.

Subclinical SVD has also been proposed to underlie the association between CKD and cognitive impairment, as patients with CKD have a tendency to develop a pattern of cognitive dysfunction with prominent deficits in executive function, attention, planning and information processing speed, which is typical for a vascular cognitive impairment profile.^17 18^

While intensive glycaemic control is known to reduce the incidence and progression of nephropathy in diabetic patients,^19^ it does not appear to have any impact on cerebral SVD.^20^

### Intracerebral haemorrhage (ICH) in CKD

CKD is prevalent in nearly one-third of patients presenting with ICH, and it is associated with much worse mortality compared with those with normal renal function.^21^ The high CKD prevalence rates in those with ICH may be attributable to their disproportionate burden of resistant hypertension, which affects one-third of patients with an eGFR of <45 mL/min/1.73 m^2^ and nearly half of patients with a urinary ACR of >300 mg/g.^22^ In addition, people with CKD (particularly black patients) appear to have a greater presence and number of CMB, further increasing their vulnerability.^23^ Mineral and bone abnormalities may also play a role since higher serum phosphate levels in these patients have been associated with an increased ICH risk, whereas low levels have been associated with an increased risk of cerebral infarction in haemodialysis patients.^24^

Compounding the problem, CKD was predictive of poorer outcomes in acute ICH in a secondary analysis of INTERACT-2, although intensive BP reduction appears to be equally effective in those with reduced eGFR compared with those with normal renal function.^25^ Patients with more advanced kidney failure had the highest risk of death or major disability at 90 days (adjusted OR 1.82, 95% CI 1.28 to 2.61).

### Vascular cognitive impairment and dementia

CKD is associated with a significant burden of cognitive impairment and dementia that increases with worsening renal function.^26^ In the Reasons for Geographic and Racial Differences in Stroke study, each 10 mL/min/1.73 m^2^ decrease in eGFR <60 mL/min/ 1.73 m^2^ was associated with an 11% increase in prevalence of cognitive dysfunction.^27^ In the haemodialysis population, the prevalence of cognitive impairment may be as high as 70%, with lower rates described in patients on peritoneal dialysis.^18 28^

Both vascular and neurodegenerative hypotheses for cognitive impairment in CKD have been proposed (table 1).^29^ Transcranial Doppler studies reveal a positive correlation between haemodynamic compromise and cognitive impairment, indicating that microvascular damage contributes to the cognitive changes observed in dementia.^30^ The pattern of cognitive change with prominent impairment of executive function is also consistent with vascular cognitive impairment.^31^

**Table 1:** Supporting evidence for the central role of cerebrovascular disease in cognitive impairment in CKD

Vascular hypothesis	Neurodegenerative hypothesis
Frequently occurring traditional risk factors, including hypertension, smoking, diabetes mellitus and ageing. Positive correlation between haemodynamic and cognitive impairment from TCD studies. Transient ‘cerebral stunning’ in haemodialysis patients associated with accelerated cognitive decline. High burden of subclinical SVD, including silent lacunar infarcts, WML and CMBs. Typical pattern of cognitive impairment with greater deficits in executive function and speed of information processing.	Chronic hypertension, highly prevalent in CKD, is known to lower the pathological threshold at which Alzheimer’s disease features manifest. Chronic cerebral inflammation due to CKD and associated vascular risk factor exposure can increase beta-amyloid production. Higher beta-amyloid levels and impaired clearance in CKD, compounded by SVD and vascular inflammation.

CKD, chronic kidney disease; CMB, cerebral microbleed; SVD, small-vessel disease; TCD, transcranial doppler; WML, white matter lesion.

Neurodegenerative mechanisms may also be augmented in CKD, and this may be partly attributable to hypertension, often comorbid in patients with CKD, and thought to potentiate Alzheimer’s pathology, lowering the threshold at which signs and symptoms manifest.^32^ In addition, high uraemic toxin concentrations of guanidine compounds present in patients with CKD are suggested to be causal as they have been found in strategic brain regions for cognition, such as the thalamus, mammillary bodies and the cerebral cortex.^33^

The Sefuri study was a population-based cohort study of 560 non-demented elderly patients that explored the relationships between CKD, subclinical cerebrovascular abnormalities and cognition using structural equation modelling, and tests of global cognitive function (Mini-Mental State Examination) and executive function (modified Stroop test).^34^ The results of this study indicate that CKD confers a risk of vascular cognitive impairment or executive dysfunction through mechanisms both dependent and independent of subclinical cerebral infarctions. This may be a consequence of long-standing untreated hypertension in early CKD as BP is positively associated with risk of vascular dementia, independent of preceding TIA or stroke.^35^

Cognitive impairment is also associated with abnormal neuroimaging in dialysis patients.^36^ In a study comparing 90 dialysis patients with 30 non-dialysis-dependent patients with CKD, cognitive impairment was found to be significantly more severe in the dialysis group. Haemodialysis patients also had more severe WMH, sulcal and ventricular atrophies, and lacunar infarcts than other patients. In all groups, higher white matter grade, ventricular grade and hippocampal atrophy were significantly associated with global cognitive impairment, with HRs of 1.80 (1.22–2.64), 1.67 (1.09–2.57) and 2.49 (1.07–5.77), respectively. Although haemodialysis patients had more severe neuroimaging features, dialysis modality showed no association with cognitive impairment.

Proteinuria shares similar associations with cognitive abnormalities. In the Systolic Blood Pressure Intervention Trial–Memory and Cognition in Decreased Hypertension substudy, patients with higher urine ACR had worse global cognitive function, executive function, memory, attention and abnormal white matter volume, such that each doubling of urine ACR had the same association with cognitive performance as being 7, 10, 6 and 14 months older, respectively.^37^

## Stroke pathophysiology in renal disease: mechanisms of susceptibility and injury

**Figure 1:**
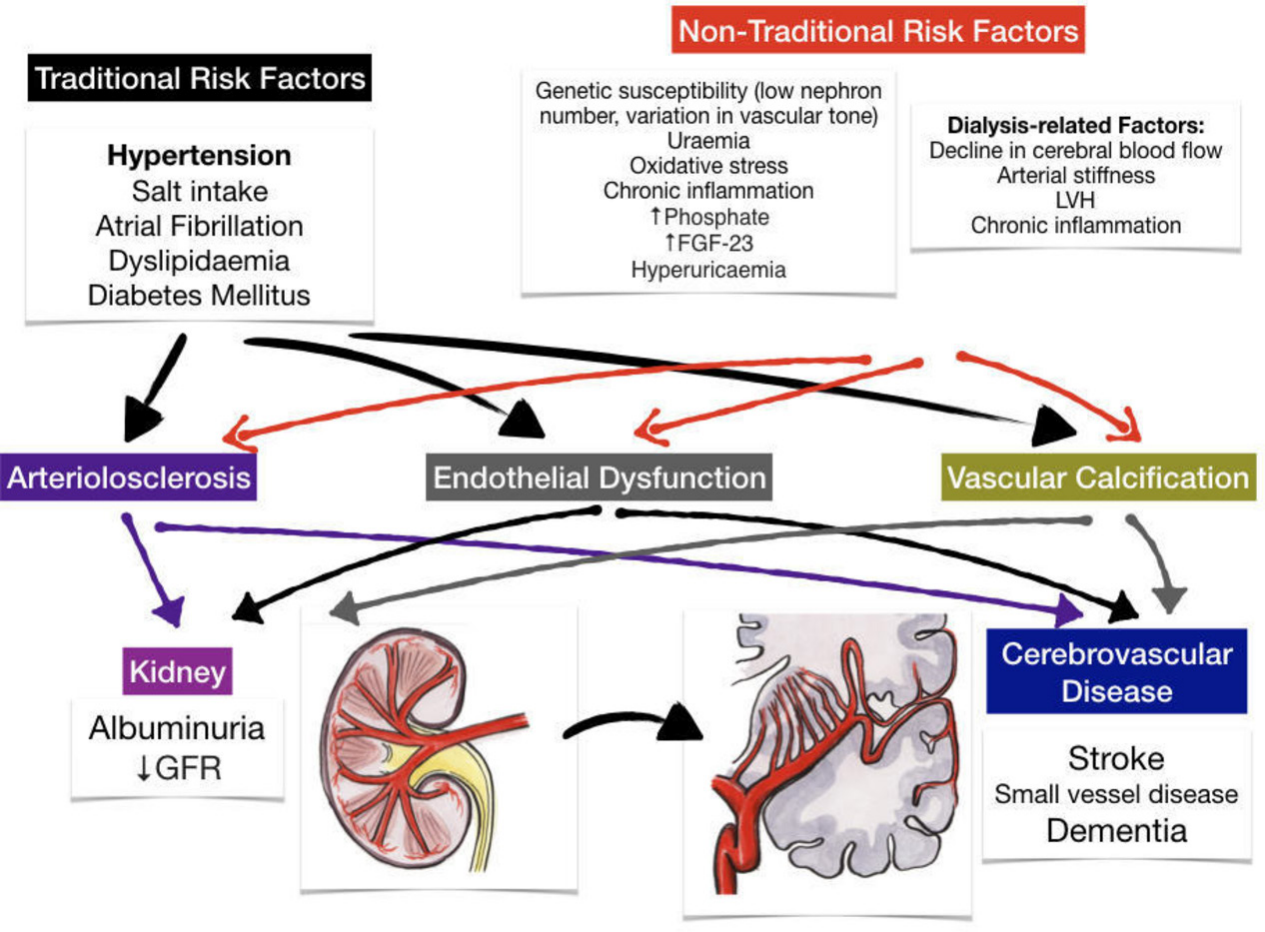
Mechanisms of susceptibility and injury in the shared pathway of renal and cerebrovascular diseases. FGF-23, fibroblast growth factor-23; GFR, glomerular filtration rate; LVH, left ventricular hypertrophy.

There is a complex relationship between CKD and cerebrovascular disease with many potential contributing mechanisms (figure 1). Some of their associations may be attributable to their shared unique pathophysiology resulting in a shared susceptibility to ‘traditional’ vascular risk factors. Putative ‘non-traditional’ causal factors directly resulting from the sequelae of renal disease may also play a role. However, there is obviously an overlap in this classification as many vascular risk factors are over-represented and accentuated in patients with renal disease. In addition, there are also some genetic or acquired conditions that may cause both CKD and stroke.

### Key shared pathophysiology and risk factors

The kidney and the brain share unique anatomical and physiological features that render them vulnerable to conventional cardiovascular risk factors such as hypertension, diabetes and smoking. They are both characterised by high blood flow rates and are dependent on local autoregulation. However, the kidney consumes twice as much oxygen as the brain and requires 20% of cardiac output to deliver a high GFR of 100–125 mL/min.^38^ We will now outline some of the proposed hypotheses that take into account the relationship between CKD and some of the major traditional vascular risk factors below.

#### Hypertension and the strain vessel hypothesis

Cardiovascular mortality doubles with every 20/10 mm Hg increment in systolic/diastolic BP in the general population.^39^ It follows then that since hypertension occurs in 67%–92% of patients with CKD,^40^ the adverse cardiovascular consequences, particularly stroke, should be expected in this group.

**Figure 2:**
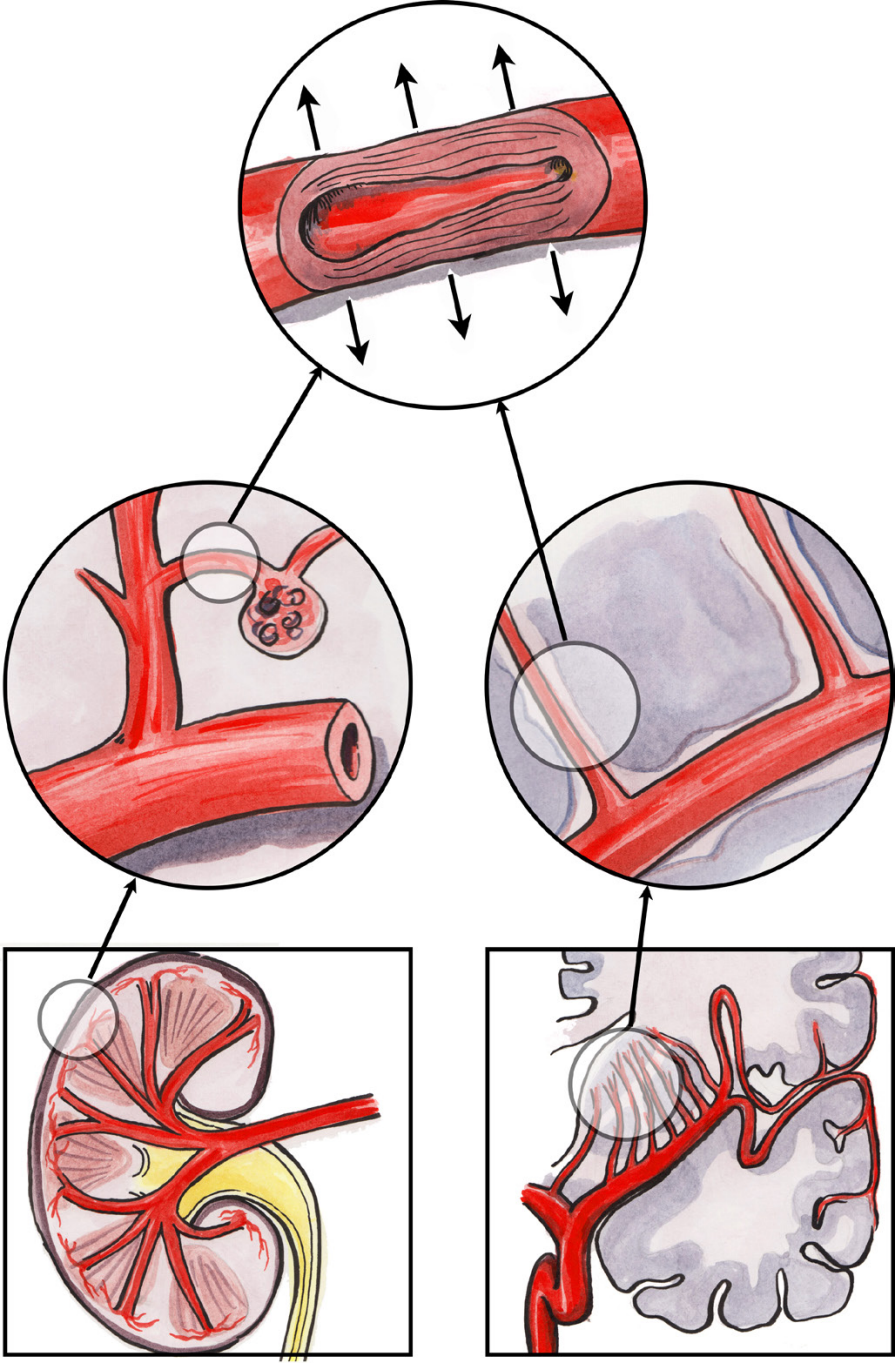
The strain vessel hypothesis: juxtamedullary afferent arterioles and cerebral perforating arteries are both exposed to high pressure and have to maintain large pressure gradients, rendering them susceptible to hypertensive injury.

The ‘strain vessel hypothesis’, based on hypertensive vascular damage, has been suggested as a possible mechanism for the association between CKD and stroke (figure 2) since both organs share similar, vulnerable microvascular vasoregulation.^41^ Both the kidney and the brain are characterised by low vascular resistance systems, allowing continuous high-volume perfusion with autoregulation ensuring constant blood flow to maintain cerebral perfusion pressure in the brain and GFR in the kidney, irrespective of fluctuations in BP.^42^

In the kidney, the juxtamedullary afferent arterioles are small, short vessels that have to maintain a strong vascular tone in order to provide a large pressure gradient in a short distance.^43^ These types of vessels are therefore referred to as ‘strain vessels’ as they are most susceptible to hypertensive renal injury. Myogenic reflexes of the smooth muscle arterioles (along with tubuloglomerular feedback) mediate the kidney’s autoregulatory response. However, with the development of hypertension, hyaline arteriolosclerosis replaces the arteriolar smooth muscle, impairing autoregulation with resultant transmission of increased systemic pressure into the glomerulus.^44^ When these afferent arterioles are damaged by hypertension, they lose their autoregulatory ability, leading to glomerular hypertension and sclerosis, progressive loss of renal function and worsening systemic hypertension.^45^ Thus, microalbuminuria, the classic manifestation of glomerular injury, may be an early marker of vascular damage of renal strain vessels.

Strain vessels also exist in the central nervous system in the form of deep perforating arteries that arise directly from large high-pressure arteries, such as anterior, middle or posterior cerebral arteries, and then penetrate into the brain tissues.^46^ Similar to the juxtamedullary afferent arterioles, these perforating arteries are also exposed to high pressures and therefore have to transmit large pressure gradients from their parent arteries to brain tissue capillaries. Analogous to renal arteriolosclerosis, lipohyalinosis may form in these strain vessels as a result of chronic hypertension, and this is also a characteristic finding in lacunar stroke. Lipohyalinosis can impair cerebral autoregulation and decrease regional cerebral blood flow, resulting in higher rates of ischaemic or haemorrhagic stroke in the areas supplied by these subcortical perforating arteries.^47 48^

Thus, there are strain vessels common to the kidney and brain that seem to be preferentially damaged by hypertension, exacerbated by the haemodynamics of large arteries, particularly arterial stiffness.^42^ Although blood flow and pressure are usually fairly constant in small vessels in the peripheral circulation, the strain vessels are exposed to pulsatile pressure and flow and are therefore more vulnerable to the deleterious effects of large-artery stiffness. However, this proposed mechanism based on shared hypertensive injury would appear to be discordant with reported epidemiological observations that stroke risk in renal disease remains after adjustment for hypertension.^6 7^

Injury of the juxtamedullary afferent arterioles (the ‘strain’ vessels) and glomeruli, of which microalbuminuria is a marker, may impair downstream medullary circulation through the vasa recta. As the medullary circulation has an important role in the mechanisms of pressure natriuresis to accomplish complete sodium balance (in response to salt ingestion),^49^ microalbuminuria may also indicate a reduction in pressure natriuresis and, therefore, salt-sensitive hypertension. This was exemplified in stroke-prone spontaneously hypertensive rats fed a high-salt diet, where the subsequent development of significant albuminuria was closely associated with vascular injuries in both the juxtamedullary nephrons and the cerebral perforating arteries.^50^ In addition to hypertensive injury, salt itself may be directly toxic to the cerebral endothelium via the production of reactive oxygen species and inflammatory cytokines from the kidney cortex.^51 52^

#### Albuminuria and generalised endothelial dysfunction (Steno hypothesis)

It has been suggested that albuminuria not only reflects localised renal damage but also is a surrogate biomarker of more generalised endothelial dysfunction associated with increased risk of vascular events such as stroke. This is the basis of the Steno hypothesis.^53^ This theory was originally derived from type 1 diabetic patients with albuminuria and proposes that albuminuria is a marker of widespread vascular damage, linking impaired vascular endothelial function with vascular leakage of albumin via glomerular barrier changes. The authors acknowledge that there is variation in the presence and the level of albuminuria between diabetic patients with corresponding differences in their cardiovascular risk, and they hypothesise that variable genetic polymorphisms of enzymes involved in the metabolism of heparin sulfate proteoglycan may be responsible for these differences. In the Framingham Heart Study, low-grade urinary albumin excretion was also associated with increased cardiovascular risk and mortality in non-hypertensive, non-diabetic middle-aged individuals.^54^ This increased risk was present even at levels well below the current diagnostic threshold for microalbuminuria. It would seem that individuals, regardless of diabetic status, are born with varying degrees of vascular function (within a physiological range) and thus excrete variable amounts of albumin.^55^ In keeping with this theory, high levels of albuminuria may already be found in young children^56^ and may reflect a normal physiological variation in endothelial function that could be associated with differential cardiovascular and renal risk at a later age.

#### Breakdown of blood–brain barrier (BBB) and glomerular barrier

BBB dysfunction with altered permeability plays an important role in both acute and chronic cerebrovascular diseases.^57^ It may contribute to SVD via the toxic leakage of fluid, proteins and other plasma constituents into the perivascular tissues with consequent oedema, arteriolar stiffening, impaired cerebral vasodilatation and oxygenation.^58^

As the BBB and the glomerular barrier in the kidney share similar structural features with tight junction complexes, they may also share similar susceptibility to mechanisms of disruption, such as hypoperfusion, ischaemic and inflammatory stimuli.^57^ The vascular endothelium, a specialised basement membrane, astrocyte foot processes and pericytes form the BBB, and this serves to restrict blood-borne substances from entering the brain.^59^ In the kidney, the glomerular filtration barrier is composed of the endothelial cell, the glomerular basement membrane and the podocyte. It determines the composition of the plasma ultrafiltrate by restricting the filtration of molecules primarily on the basis of size.^60^

Animal models of acute kidney injury and CKD have demonstrated loss of BBB integrity in the setting of uraemia,^S1, S2^ and clinical studies have shown CSF leakage of gadolinium in patients with CKD after contrast-enhanced brain MRI, consistent with impaired BBB function in these patients.^S3^ Although underlying mechanisms for this disruption are unclear, it may explain some of the association between CKD, particularly proteinuric disease, and SVD.

#### Atrial fibrillation

Patients with CKD are at high risk of AF. From the recently published Stockholm Creatinine Measurements project, 13 412 (12%) of adult patients with CKD followed up for a mean of 3.9 years developed AF with increasing incidence rates with advancing kidney disease stages: from 29.4 to 46.3 per 1000 person-years in patients with eGFR=45–60 and <30 mL/min/1.73 m^2^, respectively.^S4^ Even after adjustment for age, hypertension and cardiac disease, patients with CKD with AF were still at much higher risk of stroke and death (HR 2.00, 95% CI 1.88 to 2.14 and HR 1.76, 95% CI 1.71 to 1.82, respectively).

To compound matters, rates of prescription of anticoagulant drugs have been found to be suboptimal in people with CKD.^S5^ In addition, CKD has been associated with a left atrial (LA) thrombogenic milieu (defined as the presence of LA thrombus, dense spontaneous echo contrast or LA appendage velocity ≤25 cm/s) among patients with non-valvular AF undergoing transoesophageal echocardiography.^S6^ The prevalence of thrombogenic milieu increased with declining eGFR (4%, 18%, 36% and 86% for each group; p<0.001).

Therefore, it has been suggested to incorporate a measure of renal function in thromboembolic risk prediction scores, including the CHA_2_DS_2_–VASc-R and CHA_2_DS_2_–VAK scores.^S7, S8^ Increasing CHA_2_DS_2_–VASc-R score correlated significantly with mortality, thromboembolism and incident AF. In the CHA_2_DS_2_–VAK score, CKD was substituted for female sex, and there was enhanced discrimination of low to intermediate thromboembolic risk patients with AF in a Korean population.

#### Carotid artery disease

Although carotid artery disease is a common cause of large-artery atherosclerotic stroke in the general population, it appears to have a particularly strong association with kidney disease. In a community-based cohort study of 3364 participants, kidney function was a strong predictor of greater carotid intima–media thickness, progression of subclinical atherosclerosis, and increased fatal and nonfatal vascular events independent of traditional and non-traditional cardiovascular risk factors in multivariable analysis (HR 1.04, 95% CI 1.02 to 1.23; p=0.03 per 1 mL/min/1.73 m^2^ decrease).^S9^ This suggests that the association between CKD and carotid disease cannot be entirely explained by their common risk factors (eg, hypertension and dyslipidaemia).

It may be the case that the uraemic or the proinflammatory milieu causes CKD to impact on plaque composition, lesion stability and risk of rupture in patients with advanced carotid stenosis.^S10^ Comparison of plaque morphology was performed retrospectively on 41 patients with CKD and 56 patients with normal renal function who were undergoing carotid endarterectomy. Patients with CKD had a significantly higher percentage of total calcification (17% vs 7%, p<0.001) and unstable and ruptured plaques (83% vs 52%, p=0.001%, and 59% vs 36%, p=0.039, respectively) compared with patients with normal renal function. The content of collagenous fibres was also significantly reduced in patients with CKD. It would seem therefore that patients with CKD have a greater susceptibility to carotid disease and its cerebrovascular complications via enhanced calcification and the lower collagenous content of their carotid plaques leading to plaque instability and rupture.

A similar German study analysed metabolic and chemical parameters, along with plaque composition in relation to stroke events and renal function in patients with advanced carotid disease.^S11^ Patients with CKD again had more end-stage calcification, unstable and ruptured lesions, and lower collagenous content of their plaques. Relevant serum markers of inflammation, vascular calcification and vessel wall degradation were significantly elevated in patients with CKD compared with those with normal renal function, including enhanced levels of fibrinogen, parathyroid hormone, fetuin-A and matrix metalloproteinase-7. In accordance with this, patients with CKD experienced a higher prevalence of prior cerebrovascular events >6 months before carotid surgery (84.0% vs 26.2%, p<0.001).

Thus, CKD is not only associated with progression of carotid atherosclerosis but also with an inflammatory milieu that creates a greater propensity for unstable plaques more likely to rupture and result in cerebrovascular embolic events.

#### Dyslipidaemia

Patients with CKD can develop a secondary form of dyslipidaemia that is similar to the atherogenic dyslipidaemia of insulin-resistant patients. In addition, progressive proteinuric kidney disease can interfere with lipoprotein transport causing an increase in serum triglycerides with elevated very-low-density lipoprotein (VLDL), small dense low-density lipoprotein (LDL) particles and reduced high-density lipoprotein (HDL) cholesterol, which have significant atherogenic potential.^S12^ This is particularly prominent in patients with type 1 diabetes where serum total cholesterol, VLDL, LDL cholesterol and triglycerides all rise with increasing albuminuria.^S13^

One of the mechanisms of this ‘renal dyslipidaemia’ may be the increase in hepatic LDL synthesis that results from urinary protein loss causing upregulation of 3-hydroxy-3-methylglutaryl CoA reductase with consequent hypercholesterolaemia.^S14^ Urinary losses of the enzyme lecithin–cholesterol acyltransferase also lead to low HDL with a poor maturation of HDL-3 to cholesterol-rich HDL-2.^S15^

#### Diabetes mellitus

CKD prevalence in the diabetic population is much higher than that of the general population at 36%.^S16^ Approximately 20%–30% of both type 1 and type 2 diabetics will have moderately increased albuminuria.^S17, S18^

In addition to the associated dyslipidaemia that may result from urinary protein loss, albuminuria also appears to correlate with certain inflammatory biomarkers indicative of endothelial dysfunction, such as intercellular adhesion molecule-1. Increased levels of intercellular adhesion molecule-1, in turn, have been associated with greater burden of WMH^S19^ and progression of urinary protein loss in diabetic nephropathy.^S20^

In the Finnish Diabetic Nephropathy Study, 4083 patients with type 1 diabetes were studied.^S21^ In an adjusted analysis, the presence of microalbuminuria was associated with an increased risk of stroke with an HR of 3.2 (95% CI 1.9 to 5.6). The HRs for patients with diabetic nephropathy with macroalbuminuria and end-stage kidney disease (ESKD) were 4.9 (95% CI 2.9 to 8.2) and 7.5 (95% CI 4.2 to 13.3), respectively, with similar risks for cerebral infarction, haemorrhage and lacunar stroke. Progression of diabetic kidney disease was associated with worse stroke outcomes.^S22^

### Secondary consequences of renal dysfunction

Non-traditional CKD-related risk factors can promote cerebrovascular injury by triggering vascular injury and endothelial dysfunction. These factors include chronic inflammation, endothelial dysfunction, uraemic toxins, anaemia and mineral–bone disorder (figure 1).^4^ A recent meta-analysis of studies of risk factors in 27 465 patients with CKD identified left ventricular hypertrophy, serum albumin, phosphate, urate and haemoglobin as associated with cardiovascular events independent of traditional risk factors.^S23^

#### Uraemia

High levels of urea may contribute directly to stroke risk in patients with CKD via a reaction known as carbamylation, whereby urea dissociates to form cyanate, which, in turn, reacts irreversibly with proteins and free amino acids.^S24^ There is evidence that protein carbamylation may cause endothelial dysfunction as impaired acetylcholine-induced vasorelaxation has been described in mice with elevated urea levels. Carbamylated-oxidised LDL has also been shown to have proatherosclerotic effects on endothelial cells and macrophages, and to impair myocardial function.^S25^

The gut microbiome is also emerging as a potential source of uraemic toxins.^S26^ Disruption of the intestinal epithelial barrier due to substantial reductions in the tight junction proteins claudin-1, occludin and ZO-1 in the colonic mucosa has been observed in animal models of CKD,^S27, S28^ and this may cause gut bacterial toxins to translocate into the systemic circulation.^S29^ It is proposed that this translocation of gut toxins may promote systemic inflammation and adverse cardiovascular outcomes. In keeping with this theory, patients with CKD have different gut microbiome patterns, and uraemic toxins such as p-cresol sulfate, indoxyl sulfate and trimethylamine-N-oxide are derived from this colonic microbiome. Increased circulating levels of these uraemic solutes have been associated with a higher risk of cardiovascular morbidity and mortality in patients with ESKD.^S30^

Platelet dysfunction in the setting of uraemia may also increase the risk of haemorrhagic stroke in patients with advanced CKD.^S31^ The mechanisms of impaired platelet function in CKD include impaired platelet adhesiveness and abnormal platelet endothelial interaction. There is an imbalance between platelet agonists, ADP and serotonin, and its inhibitor, cyclic adenosine monophosphate (cAMP) resulting in an activation defect. There is also a deficiency in platelet cytoskeletal proteins that can impair motility and secretory function. Binding of both von Willebrand factor and fibrinogen to the platelet receptor glycoprotein, GPIIb/IIIa, is reduced in uraemia, leading to impaired platelet adhesion to the subendothelium.

#### Oxidative stress and chronic inflammation

The kidney is the one of the most important sources of antioxidant enzymes, such as glutathione peroxidases, and thus, levels of pro-oxidants increase with in CKD. There was a significant elevation in malondialdehyde levels (a biomarker of oxidative stress) in patients with CKD with cardiovascular disease compared with patients with CKD without cardiovascular disease, suggesting that oxidative stress potentiates atherosclerosis in CKD.^S32^

Iron therapy, frequently required to treat anaemia in advanced CKD, may also contribute to oxidative stress. Supersaturation of iron sequestration proteins such as transferrin and ferritin following the administration of intravenous iron may result in elevated levels of free iron with damaging oxidative properties.^S33^

CKD accelerates atherosclerosis by enhancing inflammation and dyslipidaemia.^S34^ Inflammation is initiated by innate immune reactions to modified lipoproteins and is perpetuated by TH1 cells that react to autoantigens from the apolipoprotein B100 protein of LDL. In addition, patients with CKD have higher levels of haemostatic factors, particularly factor VIII and von Willebrand factor, which may also augment their risk of thrombotic events.^S35^

Dialysis-dependent patients are particularly prone to chronic inflammation resulting from exogenous factors such as dialysis membranes and central venous catheters, cellular factors such as oxidative stress, tissue factors such as hypoxia and salt and fluid overload, and microbial factors such as immune dysfunction, and from the retention of uraemic toxins.^S36^

#### Mineral and bone abnormalities

Hyperphosphataemia complicates advanced CKD as a result of reduced urinary phosphate excretion, ongoing intestinal phosphate absorption and the development of secondary hyperparathyroidism.^S37^ ‘Adynamic’ bone disease may also occur with low bone turnover, decreased bone buffering capacity, higher serum calcium and higher risk of extraskeletal, particularly vascular, calcification. Arterial calcification and stiffness can lead to left ventricular hypertrophy and increased cardiovascular morbidity and mortality.^S38^

Hyperphosphataemia contributes to arterial medial calcification by inducing an osteogenic phenotype change of vascular smooth muscle cells.^S39^ Phosphate balance is regulated by fibroblast growth factor-23 (FGF-23), a protein secreted primarily by bone tissue. FGF-23 signalling requires the presence of the coreceptor Klotho.^S40^ CKD is characterised by Klotho deficiency and increased FGF-23 levels, which are independently associated with cardiovascular mortality.^S41^ In mouse models of Klotho deficiency, an accelerated ageing syndrome results with vascular calcification that resembles features of uraemia.^S42^

From a systematic review of the risk of all-cause mortality, cardiovascular mortality and cardiovascular events associated with bone–mineral abnormalities in CKD, the findings suggest a higher mortality risk with phosphate, followed by calcium and parathyroid hormone.^S43^ However, there was substantial discrepancy and heterogeneity in results with better evidence to support this association in dialysis patients than in predialysis patients.

#### Hyperuricaemia

Under normal conditions, two-thirds of the uric acid that is produced in the body is excreted in the urine and one-third is removed by the biliary tree.^S44^ CKD impairs uric acid excretion and the problem is compounded by often coexistent diuretic use, insulin resistance and increased renal vascular resistance.^S45^

Results from a meta-analysis of 15 studies with 22 571 cases of stroke and 1 042 358 participants indicate that hyperuricaemia may modestly increase the risks of both stroke incidence and mortality.^S46^ In an analysis of the Losartan Intervention for Endpoint Reduction in Hypertension Study, baseline serum uric acid was significantly associated with cardiovascular complications. Losartan's reduction of uric acid accounted for 29% of its treatment effect on the primary composite end point (cardiovascular death, myocardial infarction or stroke) compared with atenolol.^S47^

However, some propose that hyperuricaemia is simply a marker for other risk factors such as hypertension or diabetes, rather than a casual factor itself.^S48^ A discrepancy in line with this suggestion is that in patients with acute ischaemic stroke, there is a 12% increase in the odds of good clinical outcome for each milligram per decilitre increase of serum uric acid,^S49^ and the administration of uric acid therapy to patients post-thrombolysis has been associated with lower rates of early ischaemic worsening.^S50^ It has been postulated that uric acid may act as a neuroprotectant in the acute phase of stroke due to its efficient antioxidant capacity.

#### Genetic susceptibility

There is a large natural variation in nephron numbers between typical two-kidney individuals, and there is an association between nephron number and BP.^S51^ The hypothesis follows that any decrease in nephron number would be accompanied by glomerular hyperfiltration and glomerular enlargement, and would culminate in systemic hypertension. An individual’s total nephron composition, combined with a susceptibility to confounding disease factors (‘second hit’), may lead to increased risk of cardiovascular and renal diseases later in life.^S52^

Polygenic models derived using genome-wide association studies’ meta-analysis results for three kidney traits suggest that some of the risk association between CKD and cerebrovascular disease may be related to shared genetic factors. This association may vary depending on the ischaemic stroke subtypes, being particularly strong for large-vessel stroke.^S53^ Polygenic scores correlating with higher eGFR using serum creatinine were associated with lower risk of large-artery atherosclerosis. Their results also indicate a possible polygenic overlap between microalbuminuria and SVD.

COL4A1 mutations have been identified as a monogenic cause of cerebral SVD and are associated with stroke (table 2).^S54, S55^ Missense mutations of COL4A1 are also implicated in kidney disease such as hereditary angiopathy, nephropathy, aneurysms and cramps (HANAC) syndrome.^S56^ COL4A1 mutations have also been recently reported as a potential novel cause of autosomal-dominant congenital anomalies of the kidney and urinary tract (CAKUT) in humans (the most common cause of kidney disease under the age of 30).^S57^

**Table 2:** Genetic or acquired conditions causing both kidney disease and stroke

Genetically determined diseases	Features
Polycystic kidney disease	Affects 1 in 400–1000 live births; caused by mutations in PKD1 (Chr16) or PKD2 (Chr4). Renal manifestations include haematuria, hypertension, renal stones, urinary tract infection and progressive renal failure. Subarachnoid haemorrhage may be a complication of ruptured cerebral aneurysms.
COL4A1/A2-related disorders	Autosomal dominant type 4 collagen disorders causing a broad spectrum of cerebrovascular disease, including SVD and ischaemic or haemorrhagic stroke; can also be associated with hereditary nephropathy manifesting as proteinuria, haematuria, cysts or CKD.
Fabry’s disease	X-linked metabolic disorder caused by a deficiency of the enzyme α-galactosidase A. Renal manifestations such as proteinuria, isosthenuria, polyuria and polydipsia or otherwise unexplained renal insufficiency are common. TIAs and strokes occur in 25% of patients with a mean age of onset of 40 years.
Sickle cell anaemia	Autosomal recessive haemoglobinopathy. Renal complications include hyposthenuria, proteinuria, papillary/renal infarcts, hypertension and focal segmental glomerulosclerosis. Vaso-occlusive and haematological crises may result in ischaemic and haemorrhagic strokes, respectively.
Mitochondrial disorders	Renal involvement (including CKD, nephrolithiasis, nephrotic syndrome, cysts, renal tubular acidosis, Bartter-like syndrome, Fanconi syndrome, focal segmental glomerulosclerosis, tubulointerstitial nephritis and nephrocalcinosis) has been most frequently reported in patients with mitochondrial encephalomyopathy, lactic acidosis and stroke-like episode (MELAS) syndrome and Kearns-Sayre syndrome.
**Acquired conditions**	
Connective tissue disorders	
Fibromuscular dysplasia	Noninflammatory, nonatherosclerotic disorder that leads to arterial stenosis, occlusion, aneurysm, dissection and arterial tortuosity. The renal and internal carotid arteries are frequently involved.
Systemic lupus erythematosus	Up to 75% of patients may develop lupus nephritis, which can present with proteinuria, haematuria or progressive CKD. Stroke may result from secondary APS or vasculitis.
Vasculitides	
Polyarteritis nodosa	The kidneys are the most commonly affected organs, resulting in hypertension, CKD and renal infarctions. Rupture of renal arterial aneurysms can lead to perirenal haematomas. 5%–10% can have central nervous system involvement with subarachnoid haemorrhage or focal infarction.
ANCA-associated	Characterised by focal necrotising, crescentic, pauci-immune glomerulonephritis. Ischaemic infarction and intracranial haemorrhage, though rare, can be the initial presentation of ANCA-associated vasculitis and are always associated with significant morbidity
Haematological disorders	
APS	Renal disease occurs in a minority of patients with primary disease. Antiphospholipid antibodies also frequently occur in patients with lupus in whom renal disease is more frequently caused by immune deposits. Can cause ischaemic stroke in young patients along with white matter abnormalities and cognitive defects.
Plasma cell dyscrasias	Waldenström’s macroglobulinaemia is caused by a lymphoplasmacytic lymphoma in the bone marrow with an IgM monoclonal gammopathy in the blood. It can cause proteinuria, nephrotic syndrome and CKD, along with stroke due to a hyperviscosity syndrome.
Cryoglobulinaemia	A type of vasculitis caused by the deposition of circulating cryoglobulins; can be primary or associated with autoimmune diseases, malignancy or infection (particularly hepatitis C). 20% may have glomerulonephritis at presentation. TIAs and stroke can rarely occur.
Infections	
Infective endocarditis	Renal complications include bacterial infection-related immune complex-mediated glomerulonephritis, renal infarction from septic emboli and cortical necrosis. Symptomatic cerebrovascular complications, including embolic stroke or rupture of mycotic aneurysms, occur in up to 35% of patients.
HIV	CKD secondary to medication nephrotoxicity, HIV-associated nephropathy and immune complex kidney diseases are well described. Potential causes of ischaemic stroke in this setting include HIV-associated vasculopathy, opportunistic infections or neoplasia, cardioembolism and coagulopathy.
TB	Globally, TB is a common disease, with 8–10 million new cases annually and a rising incidence, particularly in regions with a high incidence of HIV infection. Classical renal TB can present with symptoms suggestive of cystitis with a sterile pyuria. Other features include calyceal distortion, ureteric strictures, bladder fibrosis and interstitial nephritis. It can also cause intracranial vasculitis resulting in stroke.
Miscellaneous	
Cocaine use	Cocaine abuse can lead to kidney injury by rhabdomyolysis, vasculitis, infarction, thrombotic microangiopathy and accelerated-phase hypertension. It may also cause stroke through a number of mechanisms, including vasospasm, cerebral vasculitis, enhanced platelet aggregation, cardioembolism and hypertensive surges associated with altered cerebral autoregulation

APS, antiphospholipid syndrome; CKD, chronic kidney disease; SVD, small-vessel disease; TB, tuberculosis; TIA, transient ischaemic attack.

Apolipoprotein E (APOE) allelles ε2 and ε4 are independent risk factors for lobar ICH, consistent with their known associations with amyloid biology.^S58^ APOE allelic variation is also a risk factor for CKD progression that is independent of established CKD risk factors such as diabetes and hypertension.^S59^

#### Dialysis-associated factors

Haemodialysis patients appear to have abnormal autonomic function as evident from their lower baroreflex sensitivity values compared with the general population^S60^ and thus are less able to tolerate acute BP dips during ultrafiltration on dialysis.^S61^ Myocardial stunning can develop during haemodialysis, resulting in or worsening any pre-existing cerebral hypoperfusion.^S62^ It has been hypothesised that such circulatory stress could produce cumulative ischaemic brain insults (‘haemodialysis-induced brain injury’).^S63^ In this UK study of a small group of haemodialysis patients (n=73), brain MRI and BP variability were examined to assess the impact of haemodialysis on brain white matter microstructure. Increased intradialytic haemodynamic instability was associated with ischaemic white matter changes, but patients who were treated with cooled dialysate (0.5°C below core body temperature) showed improved haemodynamic tolerability and demonstrated complete protection against white matter changes at 1 year.

BP control is more complex in dialysis patients, and several studies have linked lower BP to adverse outcomes, including increased mortality, especially in older or diabetic patients, or in those with pre-existing cardiac disease.^S64^ In a study of 58 haemodialysis patients, every 10 mm Hg decrease in mean arterial pressure(MAP) was associated with a 3% increase in ischaemic events, and the incidence of ischaemic events was particularly marked below an absolute MAP of 60 mm Hg.^S65^ In 23.5% of haemodialysis sessions, patients developed some evidence of transient cerebral ischaemia (measured using near-infrared spectroscopy with cerebral oxygen desaturation as a surrogate marker of ischaemia), and this intradialytic cerebral ischaemia correlated with decreased executive dysfunction 1 year later.

In a prospective observational cohort study of 97 maintenance haemodialysis patients, cerebral arterial mean flow velocity (MFV) was shown to decline significantly during dialysis, and this decline correlated with intradialytic decline in cognitive function.^S66^ Decline in MFV also correlated significantly with progression of white matter burden and cerebrovascular disease at 12 months’ follow-up. Haemodialysis is thus capable of inducing transient ‘cerebral stunning’, analogous to myocardial stunning, and may be a major mechanism of cerebral injury and accelerated cognitive decline in ESKD.

CKD is associated with increased BP variability partly due to arterial stiffness.^S67^ Greater BP variability has been shown to increase the risk of ICH in patients with CKD.^S68^ Arterial stiffness is a particularly prominent cardiovascular risk factor in haemodialysis patients. In a study examining the prognostic significance of ambulatory brachial BP, central BP, pulse wave velocity (PWV) and heart rate-adjusted augmentation index in this population, 48 hours PWV was the only vascular parameter independently associated with the primary composite cardiovascular outcome in a multivariate analysis (HR 1.579, 95% CI 1.187 to 2.102) .^S69^

### Diseases that cause both CKD and stroke

There are certain genetic and acquired conditions that can be complicated by both renal and cerebrovascular sequelae. These are outlined in table 2. Antiphospholipid syndrome (APS) is an important example, occurring either as a primary condition or in the setting of an underlying autoimmune condition such as systemic lupus erythematosus (SLE). It is defined as an autoimmune multisystem disorder characterised by arterial, venous or small-vessel thromboembolic events and/or pregnancy morbidity in the presence of persistent antiphospholipid antibodies.^S70^ Renal involvement, in the form of a thrombotic microangiopathy, may occur in as many as 25% of those with primary APS.^S71^ In patients with APS associated with SLE, renal disease may result from microthrombi and/or deposits of immune complexes.^S72^ An ischaemic stroke occurring in a young patient with renal dysfunction and no obvious risk factors for cerebrovascular disease is an important setting in which to suspect APS.

## Conclusions and future research

Patients with CKD clearly represent a high-risk group for stroke that warrant clinical prioritisation and further research focus. CKD has already been incorporated into risk prediction models such as QRISK3.^S73^ There is a need to better understand the epidemiology of cerebrovascular risk in renal disease and particularly to what extent prior hypertension may confound this relationship. There may be different mechanisms underlying the association between low GFR versus proteinuria with stroke. Better aetiological classification of stroke outcomes in future studies may help provide mechanistic insights. Preventing stroke in patients with CKD may also be a potential strategy to protect against important downstream consequences such as vascular cognitive impairment or dementia.

Additional web references are found in the online supplementary file.

10.1136/jnnp-2019-320526.supp1 Supplementary data
