# Supplementary material for: Disentangling the multiple links between renal dysfunction and cerebrovascular disease
Source: J Neurol Neurosurg Psychiatry. 2019 Sep 11;91(1):88–97. doi: 10.1136/jnnp-2019-320526 (PMC6952845; doi:10.1136/jnnp-2019-320526)
Supplement: Supplementary data [file jnnp-2019-320526supp001.pdf]

S1. Jeppsson B, Freund HR, Gimmon Z, et al. Blood-brain barrier derangement in uremic encephalopathy. *Surgery* 1982;92(1):30-5.

S2. Liu M, Liang Y, Chigurupati S, et al. Acute kidney injury leads to inflammation and functional changes in the brain. *J Am Soc Nephrol.* 2008;19(7):1360-70. doi: 10.1681/asn.2007080901

S3. Rai AT, Hogg JP. Persistence of gadolinium in CSF: a diagnostic pitfall in patients with end-stage renal disease. *AJNR Am J Neuroradiol.* 2001;22(7):1357-61.

S4. Carrero JJ, Trevisan M, Sood MM, et al. Incident Atrial Fibrillation and the Risk of Stroke in Adults with Chronic Kidney Disease: The Stockholm CREAtinine Measurements (SCREAM) Project. *Clin J Am Soc Nephrol.* 2018;13(9):1314-20. doi: 10.2215/cjn.04060318

S5. Bassand JP, Accetta G, Al Mahmeed W, et al. Risk factors for death, stroke, and bleeding in 28,628 patients from the GARFIELD-AF registry: Rationale for comprehensive management of atrial fibrillation. *PloS one* 2018;13(1):e0191592. doi: 10.1371/journal.pone.0191592

S6. Kizawa S, Ito T, Akamatsu K, et al. Chronic Kidney Disease as a Possible Predictor of Left Atrial Thrombogenic Milieu Among Patients with Nonvalvular Atrial Fibrillation. *Am J Cardiol.* 2018 doi: 10.1016/j.amjcard.2018.08.058

S7. Parsons C, Cha S, Shen WK, et al. Usefulness of the Addition of Renal Function to the CHA2DS2-VASc Score as a Predictor of Thromboembolism and Mortality in Patients Without Atrial Fibrillation. *Am J Cardiol.* 2018;122(4):597-603. doi: 10.1016/j.amjcard.2018.04.049

S8. Cha MJ, Cho Y, Oh IY, et al. Validation of Conventional Thromboembolic Risk Factors in a Korean Atrial Fibrillation Population- Suggestion for a Novel Scoring System, CHA2DS2-VAK. *Circ J.* 2018 doi: 10.1253/circj.CJ-18-0218

S9. Desbien AM, Chonchol M, Gnahn H, et al. Kidney function and progression of carotid intima-media thickness in a community study. *Am J Kidney Dis.* 2008;51(4):584-93. doi: 10.1053/j.ajkd.2007.11.026

S10. Pelisek J, Assadian A, Sarkar O, et al. Carotid plaque composition in chronic kidney disease: a retrospective analysis of patients undergoing carotid endarterectomy. *Eur J Vasc Endovasc Surg.* 2010;39(1):11-6. doi: 10.1016/j.ejvs.2009.09.024

S11. Pelisek J, Hahntow IN, Eckstein HH, et al. Impact of chronic kidney disease on carotid plaque vulnerability. *J Vasc Surg.* 2011;54(6):1643-9. doi: 10.1016/j.jvs.2011.05.049

S12. Vaziri ND. Molecular mechanisms of lipid disorders in nephrotic syndrome. *Kidney international* 2003;63(5):1964-76. doi: 10.1046/j.1523-1755.2003.00941.

S13. Jenkins AJ, Lyons TJ, Zheng D, et al. Lipoproteins in the DCCT/EDIC cohort: associations with diabetic nephropathy. *Kidney Int.* 2003;64(3):817-28. doi: 10.1046/j.1523-1755.2003.00164.

S14. Vaziri ND, Sato T, Liang K. Molecular mechanisms of altered cholesterol metabolism in rats with spontaneous focal glomerulosclerosis. *Kidney Int.* 2003;63(5):1756-63. doi: 10.1046/j.1523-1755.2003.00911.

S15. Vaziri ND, Liang K, Parks JS. Acquired lecithin-cholesterol acyltransferase deficiency in nephrotic syndrome. *Am J Physiol Renal Physiol.* 2001;280(5):F823-8. doi: 10.1152/ajprenal.2001.280.5.F823

S16. System USRD. 2018 USRDS annual data report: Epidemiology of kidney disease in the United States. . *National Institutes of Health, National Institute of Diabetes and Digestive and Kidney Diseases, Bethesda, MD* 2018:A4.

S17. Orchard TJ, Dorman JS, Maser RE, et al. Prevalence of complications in IDDM by sex and duration. Pittsburgh Epidemiology of Diabetes Complications Study II. *Diabetes* 1990;39(9):1116-24.
S18. Adler AI, Stevens RJ, Manley SE, et al. Development and progression of nephropathy in type 2 diabetes: the United Kingdom Prospective Diabetes Study (UKPDS 64). *Kidney Int.* 2003;63(1):225-32. doi: 10.1046/j.1523-1755.2003.00712.x
S19. Shoamanesh A, Preis SR, Beiser AS, et al. Inflammatory biomarkers, cerebral microbleeds, and small vessel disease: Framingham Heart Study. *Neurology* 2015;84(8):825-32. doi: 10.1212/wnl.0000000000001279
S20. Lin J, Glynn RJ, Rifai N, et al. Inflammation and progressive nephropathy in type 1 diabetes in the diabetes control and complications trial. *Diabetes care* 2008;31(12):2338-43. doi: 10.2337/dc08-0277
S21. Hagg S, Thorn LM, Putaala J, et al. Incidence of stroke according to presence of diabetic nephropathy and severe diabetic retinopathy in patients with type 1 diabetes. *Diabetes care* 2013;36(12):4140-6. doi: 10.2337/dc13-0669
S22. Hagg-Holmberg S, Thorn LM, Forsblom CM, et al. Prognosis and Its Predictors After Incident Stroke in Patients With Type 1 Diabetes. *Diabetes care* 2017;40(10):1394-400. doi: 10.2337/dc17-0681
S23. Major RW, Cheng MRI, Grant RA, et al. Cardiovascular disease risk factors in chronic kidney disease: A systematic review and meta-analysis. *PloS one* 2018;13(3):e0192895. doi: 10.1371/journal.pone.0192895
S24. Kalim S, Karumanchi SA, Thadhani RI, et al. Protein carbamylation in kidney disease: pathogenesis and clinical implications. *Am J Kidney Dis.* 2014;64(5):793-803. doi: 10.1053/j.ajkd.2014.04.034
S25. Apostolov EO, Ok E, Burns S, et al. Carbamylated-oxidized LDL: proatherosclerotic effects on endothelial cells and macrophages. *J Atheroscler Thromb.* 2013;20(12):878-92.
S26. Ramezani A, Raj DS. The gut microbiome, kidney disease, and targeted interventions. *J Am Soc Nephrol.* 2014;25(4):657-70. doi: 10.1681/asn.2013080905
S27. Vaziri ND, Yuan J, Rahimi A, et al. Disintegration of colonic epithelial tight junction in uremia: a likely cause of CKD-associated inflammation. *Nephrol Dial Transplant.* 2012;27(7):2686-93. doi: 10.1093/ndt/gfr624
S28. Lau WL, Kalantar-Zadeh K, Vaziri ND. The Gut as a Source of Inflammation in Chronic Kidney Disease. *Nephron* 2015;130(2):92-8. doi: 10.1159/000381990
S29. Szeto CC, Kwan BC, Chow KM, et al. Endotoxemia is related to systemic inflammation and atherosclerosis in peritoneal dialysis patients. *Clin J Am Soc Nephrol.* 2008;3(2):431-6. doi: 10.2215/cjn.03600807
S30. Shafi T, Meyer TW, Hostetter TH, et al. Free Levels of Selected Organic Solutes and Cardiovascular Morbidity and Mortality in Hemodialysis Patients: Results from the Retained Organic Solutes and Clinical Outcomes (ROSCO) Investigators. *PloS one* 2015;10(5):e0126048. doi: 10.1371/journal.pone.0126048
S31. Kaw D, Malhotra D. Platelet dysfunction and end-stage renal disease. *Semin Dial.* 2006;19(4):317-22. doi: 10.1111/j.1525-139X.2006.00179.
S32. Boaz M, Matas Z, Biro A, et al. Serum malondialdehyde and prevalent cardiovascular disease in hemodialysis. *Kidney Int.* 1999;56(3):1078-83. doi: 10.1046/j.1523-1755.1999.00613.
S33. Zager RA. Parenteral iron compounds: potent oxidants but mainstays of anemia management in chronic renal disease. *Clin J Am Soc Nephrol.* 2006;1 Suppl 1:S24-31. doi: 10.2215/cjn.01410406

S34. Gistera A, Hansson GK. The immunology of atherosclerosis. *Nat Rev Nephrol.* 2017;13(6):368-80. doi: 10.1038/nrneph.2017.51
S35. Huang MJ, Wei RB, Wang Y, et al. Blood coagulation system in patients with chronic kidney disease: a prospective observational study. *BMJ open* 2017;7(5):e014294. doi: 10.1136/bmjopen-2016-014294
S36. Cobo G, Lindholm B, Stenvinkel P. Chronic inflammation in end-stage renal disease and dialysis. *Nephrol Dial Transplant.* 2018;33(suppl_3):iii35-iii40. doi: 10.1093/ndt/gfy175
S37. Hruska KA, Mathew S, Lund R, et al. Hyperphosphatemia of chronic kidney disease. *Kidney Int.* 2008;74(2):148-57. doi: 10.1038/ki.2008.130
S38. Lau WL, Ix JH. Clinical detection, risk factors, and cardiovascular consequences of medial arterial calcification: a pattern of vascular injury associated with aberrant mineral metabolism. *Semin Nephrol.* 2013;33(2):93-105. doi: 10.1016/j.semnephrol.2012.12.011
S39. Jono S, McKee MD, Murry CE, et al. Phosphate regulation of vascular smooth muscle cell calcification. *Circ Res.* 2000;87(7):E10-7.
S40. Urakawa I, Yamazaki Y, Shimada T, et al. Klotho converts canonical FGF receptor into a specific receptor for FGF23. *Nature* 2006;444(7120):770-4. doi: 10.1038/nature05315
S41. Gutierrez OM, Mannstadt M, Isakova T, et al. Fibroblast growth factor 23 and mortality among patients undergoing hemodialysis. *N Engl J Med.* 2008;359(6):584-92. doi: 10.1056/NEJMoa0706130
S42. Kuro-o M, Matsumura Y, Aizawa H, et al. Mutation of the mouse klotho gene leads to a syndrome resembling ageing. *Nature* 1997;390(6655):45-51. doi: 10.1038/36285
S43. Covic A, Kothawala P, Bernal M, et al. Systematic review of the evidence underlying the association between mineral metabolism disturbances and risk of all-cause mortality, cardiovascular mortality and cardiovascular events in chronic kidney disease. *Nephrol Dial Transplant.* 2009;24(5):1506-23. doi: 10.1093/ndt/gfn613
S44. Hyndman D, Liu S, Miner JN. Urate Handling in the Human Body. *Curr Rheumatol Rep.* 2016;18(6):34. doi: 10.1007/s11926-016-0587-7
S45. Jalal DI, Chonchol M, Chen W, et al. Uric acid as a target of therapy in CKD. *Am J Kidney Dis.* 2013;61(1):134-46. doi: 10.1053/j.ajkd.2012.07.021
S46. Li M, Hou W, Zhang X, et al. Hyperuricemia and risk of stroke: a systematic review and meta-analysis of prospective studies. *Atherosclerosis* 2014;232(2):265-70. doi: 10.1016/j.atherosclerosis.2013.11.051
S47. Hoieggen A, Alderman MH, Kjeldsen SE, et al. The impact of serum uric acid on cardiovascular outcomes in the LIFE study. *Kidney Int.* 2004;65(3):1041-9. doi: 10.1111/j.1523-1755.2004.00484.
S48. Culleton BF, Larson MG, Kannel WB, et al. Serum uric acid and risk for cardiovascular disease and death: the Framingham Heart Study. *Ann Intern Med.* 1999;131(1):7-13.
S49. Chamorro A, Obach V, Cervera A, et al. Prognostic significance of uric acid serum concentration in patients with acute ischemic stroke. *Stroke* 2002;33(4):1048-52.
S50. Amaro S, Laredo C, Renu A, et al. Uric Acid Therapy Prevents Early Ischemic Stroke Progression: A Tertiary Analysis of the URICO-ICTUS Trial (Efficacy Study of Combined Treatment With Uric Acid and r-tPA in Acute Ischemic Stroke). *Stroke* 2016;47(11):2874-76. doi: 10.1161/strokeaha.116.014672

S51. Brenner BM, Garcia DL, Anderson S. Glomeruli and blood pressure. Less of one, more the other? *Am J Hypertens.* 1988;1(4 Pt 1):335-47.
S52. Wang X, Garrett MR. Nephron number, hypertension, and CKD: physiological and genetic insight from humans and animal models. *Physiol Genomics.* 2017;49(3):180-92. doi: 10.1152/physiolgenomics.00098.2016
S53. Holliday EG, Traylor M, Malik R, et al. Polygenic overlap between kidney function and large artery atherosclerotic stroke. *Stroke* 2014;45(12):3508-13. doi: 10.1161/strokeaha.114.006609
S54. Lanfranconi S, Markus HS. COL4A1 mutations as a monogenic cause of cerebral small vessel disease: a systematic review. *Stroke* 2010;41(8):e513-8. doi: 10.1161/strokeaha.110.581918
S55. Malik R, Rannikmae K, Traylor M, et al. Genome-Wide Meta-analysis identifies three novel loci associated with stroke. *Ann Neurol.* 2018 doi: 10.1002/ana.25369
S56. Plaisier E, Ronco P. COL4A1-Related Disorders. In: Adam MP, Ardinger HH, Pagon RA, et al., eds. GeneReviews((R)). Seattle (WA): University of Washington, Seattle. University of Washington, Seattle.
S57. Kitzler T KC, Schneider R, Connaughton DM, Majmundar AJ, Mann N, Nakayama M, Shril S, Hildebrandt F. COL4A1 Mutations as a Potential Novel Cause of Autosomal-Dominant CAKUT in Humans. *J Am Soc Nephrol.* 2018;29:302.
S58. Biffi A, Sonni A, Anderson CD, et al. Variants at APOE influence risk of deep and lobar intracerebral hemorrhage. *Ann Neurol.* 2010;68(6):934-43. doi: 10.1002/ana.22134
S59. Hsu CC, Kao WH, Coresh J, et al. Apolipoprotein E and progression of chronic kidney disease. *JAMA.* 2005;293(23):2892-9. doi: 10.1001/jama.293.23.2892
S60. Chesterton LJ, Sigrist MK, Bennett T, et al. Reduced baroreflex sensitivity is associated with increased vascular calcification and arterial stiffness. *Nephrol Dial Transplant.* 2005;20(6):1140-7. doi: 10.1093/ndt/gfh808
S61. Chesterton LJ, Selby NM, Burton JO, et al. Categorization of the hemodynamic response to hemodialysis: the importance of baroreflex sensitivity. *Hemodial Int.* 2010;14(1):18-28. doi: 10.1111/j.1542-4758.2009.00403.
S62. Burton JO, Jefferies HJ, Selby NM, et al. Hemodialysis-induced cardiac injury: determinants and associated outcomes. *Clin J Am Soc Nephrol.* 2009;4(5):914-20. doi: 10.2215/cjn.03900808
S63. Eldehni MT, Odudu A, McIntyre CW. Randomized clinical trial of dialysate cooling and effects on brain white matter. *J Am Soc Nephrol.* 2015;26(4):957-65. doi: 10.1681/asn.2013101086
S64. Myers OB, Adams C, Rohrscheib MR, et al. Age, race, diabetes, blood pressure, and mortality among hemodialysis patients. *J Am Soc Nephrol.* 2010;21(11):1970-8. doi: 10.1681/asn.2010010125
S65. MacEwen C, Sutherland S, Daly J, et al. Relationship between Hypotension and Cerebral Ischemia during Hemodialysis. *J Am Soc Nephrol.* 2017;28(8):2511-20. doi: 10.1681/asn.2016060704
S66. Findlay MD, Dawson J, Dickie DA, et al. Investigating the Relationship between Cerebral Blood Flow and Cognitive Function in Hemodialysis Patients. *J Am Soc Nephrol.* 2019;30(1):147-58. doi: 10.1681/asn.2018050462
S67. Tanner RM, Shimbo D, Dreisbach AW, et al. Association between 24-hour blood pressure variability and chronic kidney disease: a cross-sectional analysis of African Americans participating in the Jackson heart study. *BMC Nephrol.* 2015;16:84. doi: 10.1186/s12882-015-0085-6

S68. Chang TI, Tabada GH, Yang J, et al. Visit-to-visit variability of blood pressure and death, end-stage renal disease, and cardiovascular events in patients with chronic kidney disease. *J Hypertens.* 2016;34(2):244-52. doi: 10.1097/hjh.0000000000000779

S69. Sarafidis PA, Loutradis C, Karpetas A, et al. Ambulatory Pulse Wave Velocity Is a Stronger Predictor of Cardiovascular Events and All-Cause Mortality Than Office and Ambulatory Blood Pressure in Hemodialysis Patients. *Hypertension* 2017;70(1):148-57. doi: 10.1161/hypertensionaha.117.09023

S70. Miyakis S, Lockshin MD, Atsumi T, et al. International consensus statement on an update of the classification criteria for definite antiphospholipid syndrome (APS). *J Thromb Haemost.* 2006;4(2):295-306. doi: 10.1111/j.1538-7836.2006.01753.x

S71. Amigo MC, Garcia-Torres R, Robles M, et al. Renal involvement in primary antiphospholipid syndrome. *J Rheumatol.* 1992;19(8):1181-5.

S72. Daugas E, Nochy D, Huong DL, et al. Antiphospholipid syndrome nephropathy in systemic lupus erythematosus. *J Am Soc Nephrol.* 2002;13(1):42-52.

S73. Hippisley-Cox J, Coupland C, Brindle P. Development and validation of QRISK3 risk prediction algorithms to estimate future risk of cardiovascular disease: prospective cohort study. *BMJ.* 2017;357:j2099. doi: 10.1136/bmj.j2099
